# An Analytical Evaluation of the Synergistic Effect on Biodiesel Oxidation Stability Promoted by Binary and Ternary Blends Containing Multifunctional Additives

**DOI:** 10.1155/2019/6467183

**Published:** 2019-01-01

**Authors:** Silvia S. Yahagi, Ana C. Roveda, Adrielli T. Sobral, Ivan P. Oliveira, Anderson R. L. Caires, Roberto S. Gomes, Magno A. G. Trindade

**Affiliations:** ^1^Faculty of Exact Sciences and Technology, Federal University of Grande Dourados, Highway Dourados-Itahum, Km 12, P.O. Box 364, 79804-970 Dourados, MS, Brazil; ^2^Synthesis and Molecular Modification Laboratory, Faculty of Exact Sciences and Technology, Federal University of Grande Dourados, Rodovia Dourados-Itahum, Km 12, 79804-970 Dourados, MS, Brazil; ^3^Institute of Biomedical Sciences, University of São Paulo, Av. Prof. Lineu Prestes, 1524, 05508-900 São Paulo, SP, Brazil; ^4^Optics and Photonics Group, Institute of Physics, Federal University of Mato Grosso do Sul, P.O. Box 549, 79070900 Campo Grande, MS, Brazil

## Abstract

The antioxidant potential of a novel additive, named maleimide* p*-CH_3_, and the synergistic effect on biodiesel stabilization when combined with a traditional synthetic antioxidant (e.g., propyl gallate (PG)) as well as alternative additives (e.g., alizarin (ALZ) and citric acid (CA)) were investigated. The additives were combined in binary and/or ternary blends at low concentrations and their effectiveness against soybean biodiesel oxidation in the absence and presence of metal was tested. The effectiveness of binary and ternary blends was also evaluated by the Rancimat® method and compared with total acid number (TAN). The results showed that the combinations presented synergetic effects and were effective in stabilizing biodiesel in accordance with the minimum requirements of EN 14214 and also revealed that the mixture containing PG and* p*-CH_3_, even at low concentrations, can be successfully applied for biodiesel preservation. In summary, we report that the biodiesel stability can be obtained by using a reduced amount of additives, suggesting that biodiesel shelf life can be improved in association with a cost reduction when compared to the use of conventional antioxidants.

## 1. Introduction

Environmental issues associated with the use of fuels obtained from petroleum have been widely discussed [1, 2], especially due to the harmful effects caused by the emissions of carbon dioxide, nitric oxides, and sulphur compounds [3]. In addition, it is well known that petroleum is a limited resource as it is expected to end up in the near future [4]. In face that, alternative resources of energy such as wind energy, solar energy, geothermal, and biofuels have been searched [5, 6]. Among the biofuels, biodiesel has gained attention as an environmental friendly fuel, composed by fatty acids methyl esters (FAME) obtained from renewable feedstock, which can partially or totally substitute the petrodiesel during engine operation [7, 8]. In order to reduce environmental impacts, a number of countries had introduced a mandatory addition of biodiesel to fossil diesel, for example, the compulsory addition of 30% biodiesel to the diesel fuel regulated by Czech Republic and Slovakia and 20% by United States and Canada as well as 5% in the European Union and Argentina [9]. In Brazil the current addition is 10%; however, the government is revising this regulation and authorized a schedule of increase of one percentage point per year, from 2020 until 2023, of which the goal is to achieve an addition of 15% of biodiesel in the diesel-biodiesel blend.

Among all the advantages associated with biodiesel, low oxidative stability has been one of the main drawbacks to its consolidation in the world energy matrix and consequently one of the most important challenges for the development of this area [10]. Biodiesel oxidation has its origins in the unsaturated fatty acids carbon chains which are easily oxidized, forming primary degraded compounds (e.g., peroxides and hydroperoxides) and then secondary compounds (such as ketones, aldehydes, and carboxylic acids) [11]. As a consequence, biodiesel acidity is increased after the oxidation process which may lead to corrosion of metallic materials such as storage reservoirs as well as several parts of the engine due to the increase of its corrosive characteristics [12]. Different environmental conditions may induce an acceleration of alkyl esters carbon chains oxidation in which the main causes are luminosity, humidity, high temperatures, metal contaminants, and oxygen presence (O_2_ singlet and triplet forms) [12, 13]. During biodiesel degradation induced by all of these oxidation inductors, a formation of radicals from alkyl chains is expected. Based on that, biodiesel stability can be managed by additives, aiming to retard oxidation and guarantee the quality of biodiesel, as the formed radicals are stronger catalysts and maybe neutralized with addition of synthetic or natural antioxidant [14–16].

Synthetic antioxidants are effective and widely applied to improve the oxidation stability of biodiesel [17]. However, depending on the biodiesel composition, especially the number of unsaturations in carbon chains, a high amount of these additives is required to obtain the mandatory specifications requested by the standards agencies, increasing the production costs [10]. In this sense, an efficient antioxidant additive must improve the shelf life of biodiesel without change any other properties of this fuel. The efficiency of a given antioxidant is dependent on its chemical structure and its power as free radical scavenger.

**Figure 1:**
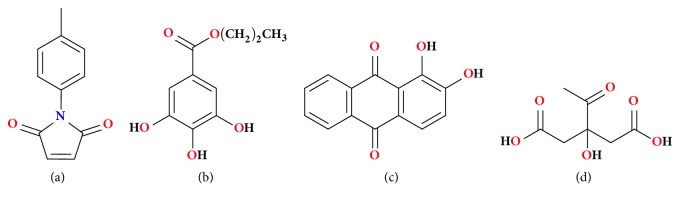
Chemical structures of (a) maleimide (*p*-CH_3_), (b) propyl gallate (PG), (c) alizarin (ALZ), and (d) citric acid (CA).

Interestingly, it is worth pointing out that even when added at high concentrations, conventional antioxidants have not been efficient against biodiesel degradation induced by heating or metals [18, 19], being a critical limitation that must be overcome for new additives. For instance, it is known that metals are responsible to catalyse the oxidation and polymerization reactions of unsaturated fatty acids. Aiming to improve the antioxidative action of additives, synergistic combinations of different antioxidants with other compounds can be applied to biodiesel stabilization and reduce the amount of used additives. Our research group recently reported that the use of anthraquinone dyes in combination with synthetic antioxidants can significantly improve the biodiesel oxidative stability [9, 10, 12, 20, 21]. The results revealed the existence of a positive synergism, in which the combinations were more efficient when compared to the individual additives, providing greater stability to the biodiesel even in presence of prooxidative metals [9, 10, 12, 20, 21]. In the present study, we evaluate the antioxidant potential of a novel additive, named maleimide* p*-CH_3_, and its combination with a traditional synthetic antioxidant (e.g., propyl gallate, PG), as well as the alternative additives (e.g., alizarin, ALZ, and citric acid, CA) in order to have a mixture of multifunctional additives. The additives were combined at low concentrations and their effectiveness against soybean biodiesel oxidation in the absence and presence of metals was evaluated. The chemical structures of all investigated compounds are shown in Figure 1.

## 2. Materials and Methods

### 2.1. Chemicals and Instrumentation

Propyl gallate (PG, 97%), citric acid (CA, 99.5%), alizarin (ALZ, 97%), and copper (II) nitrate trihydrate (99.9%) were purchased from Sigma-Aldrich (São Paulo, Brazil). All chemicals—ethanol (anhydrous, 99.9%), methanol (anhydrous, 99.5%), sodium hydroxide (pallets, 99.5%), chloroform (anhydrous, 99.5%), sodium sulphate (anhydrous, 99%), sodium chloride (anhydrous, 99%), potassium iodide (anhydrous, 99%), starch solution (1.0% w/v), potassium permanganate (anhydrous, 99%), sodium thiosulphate (anhydrous, 99%), and acetic acid (anhydrous, 99%)—were of analytical grade and purchased from Vetec (Rio de Janeiro, Brazil).

Highly purified water (R ≥ 18.2 MΩ cm) was obtained in an OS 10 LTXE reverse osmosis water purifier (Gehaka®, São Paulo, Brazil) and was used to prepare working solutions. A magnetic stirrer with heating (Fisatom, model 752A) was used during soybean biodiesel production. Ultrasound (Unique®, Model USC 1400A) was used to promote a complete solubilization of the samples. Accelerated biodiesel degradation was conducted in a laboratory oven (Ethik technology, São Paulo, Brazil). The induction period of biodiesel samples was performed in a Rancimat® instrument (model 893, Metrohm, Switzerland).

^1^H and ^13^C NMR spectra were obtained at 300 and 75 MHz, respectively, using a Bruker Avance DPX-300 spectrometer. Chemical shifts are reported relative to TMS; coupling constants are provided in hertz.

### 2.2. Synthesis of* p*-Methyl-aryl-maleimide

A solution of maleic anhydride (5.0 mmol),* p*-methylaniline (10.0 mmol), in ether was stirred for 3 hours at room temperature. The precipitate was filtered, washed with cold water, and dried at room temperature. Then, to a flask containing acetic anhydride (36.1 mmol) and sodium acetate (4 mmol) the precipitate without further purification was added and warmed under steam bath for 1.0 hour. The reaction mixture was transferred to a Becker containing water and ice, then the precipitate was filtered and recrystallized in ethanol/H_2_O (1:1), yield = 74%.

^1^H-NMR (CDCl_3_, 300 MHz) *δ* (ppm): 2.36 (*s*, 3H); 6.82 (*s*, 2H); 7.18 (*d*, 2H); 7.26 (*d*, 2H). ^13^C-NMR (CDCl_3_, 75 MHz) *δ* (ppm): 21.1 (CH_3_); 126.0 (CH); 128.5 (C); 129.8 (CH); 134.2 (CH); 138.1 (C); 169.7 (C).

### 2.3. Soybean Biodiesel Production

Biodiesel was obtained via alkaline transesterification of soybean oil using potassium hydroxide (KOH) as the basic catalyst (1.5% w/w) and a 6:1 molar ratio of methanol/oil at a temperature of 45°C under stirring constant for 90 min. For the separation of biodiesel from glycerine and undesirable components, the mixture was transferred to a settling funnel, where it remained for approximately 24 hours. The biodiesel was separated from the by-products by decantation and washed three times with 50 mL ultrapure water and then with 20 mL of saturated sodium chloride solution. After that, the biodiesel was filtered in the presence of anhydrous sodium sulphate (Na_2_SO_4_).

### 2.4. Additives and Metal Addition into Biodiesel

Combination of different quantities of antioxidant compounds (PG, ALZ, and CA), maleimide, and copper (II) nitrate trihydrate was added to the soybean biodiesel samples at different concentrations (mg·kg^−1^) as presented in Tables 1, 2, and 3. The nomenclature used to differentiate the various doped and nondoped samples is also shown in Tables 1–3.

### 2.5. Evaluation of Oxidative Stability

The induction period (IP) was obtained by Rancimat method according to EN 14112 [22], determining the biodiesel oxidative stability. For this, 3.00 g of sample was placed into heating block at 110°C under constant air flow (10 L·h^−1^) and then to a vessel containing 50 ml of distilled water. The IP was determined by applying the second derivative to the conductivity* versus* time curves as in Rancimat method there is an abrupt increase on the conductivity related to the formation of volatile products, small organic acids, generated by the degradation of the biodiesel. All analyses were performed in duplicate.

### 2.6. Total Acid Number Quantification

For quantification of the total acid number (TAN), 5.0 g of each biodiesel sample shown in Tables 1–3 was subjected to continuous heating in a laboratory oven at 85°C for 62 h. During accelerated oxidation, aliquots were periodically extracted every 3 hours to be analysed and monitor the degree of oxidation through the TAN. The TAN determination was performed according to Roveda et al. [9, 12]. 2.00 g (± 0.01 g) of soybean biodiesel was weighed and dissolved in 20 mL of water/ethanol (2:3 v/v) solution. A solution of phenolphthalein 1.0 % (w/v) was then added, and then the titration was carried out with a 0.01 mol·L^−1^ KOH hydroalcoholic solution (40:60 v/v, water/ethanol) until the development of the pink colour persisted for at least 30 s.

## 3. Results and Discussion

### 3.1. Evaluation the Effectiveness of the Individual Additives

The influence of individual additives on biodiesel stability, according Rancimat method, is shown in Table 4. The results show that pure biodiesel (P) had an induction period of 3.76 ± 0.25 h, in accordance with previous results reported in the literature [10, 12, 18]. Differently, for the sample P-A1, there is only copper addition in its composition, and the IP drops to 0.20 ± 0.05 h. These results demonstrated that the addition of Cu(II) induced the reduction of biodiesel stability due to the high catalytic activity associated with metal [12]. Additionally, the results also revealed that the samples containing maleimide (*p*-CH_3_) at concentrations of 25, 50, and 100 mg·kg^−1^ (codes P-B1, P-B2 and P-B3, respectively) presented low IP values, indicating that* p*-CH_3_ used alone has a very low potential for biodiesel protection. On the contrary, the isolated addition of PG into biodiesel samples resulted in a considerable increase of IP, mainly when added at 100 and 150 mg·kg^−1^. However, it was not effective at high concentration (200 mg·kg^−1^). With regard to ALZ and CA, it was verified that both additives promoted an increase in IP values (e.g., of 3.40 ± 0.05 and 4.99 ± 0.05, respectively), confirming their antioxidant activities on biodiesel even at low concentrations (20 mg·kg^−1^).

### 3.2. Evaluation the Effectiveness of the Binary Additive Blends

Table 5 shows the results obtained from binary combinations of the antioxidants with the addition of copper as a prooxidative metal. In some samples (i.e., CB-A1, CB-A2 and CB-A4), the IP values obtained was inferior to that presented by the PG used as individual additive: for example, 4.34 ± 0.01 (CB-A1) < 5.04 ± 0.15 (P-C1); 6.23 ± 0.13 (CB-A2) < 7.49 ± 0.18 (P-C3); 1.92 ± 0.01 (CB-A4) < 3.40 ± 0.05 (P-C4), respectively. The possible reason is the degradation of* p*-CH_3_, leading to a reduction of the antioxidant activity, which in turn leaves the biodiesel susceptible to the oxidative process in the presence of prooxidative metal.

For samples containing* p*-CH_3_ at concentrations of 50 and 100 mg·kg^−1^ in combination with PG at 50 and 100 mg kg^−1^ (Table 5, CB-B1, CB-B2, and CB-C1, CB-C2, CB-C3), the IP values were lower than those obtained when the PG was used as individual additive (samples code: P-C1, P-C2 and P-C3, Table 4). This is indicative of the absence of synergistic effect when these additives are used in combination, even increasing the concentration of the* p*-CH_3_ up to 100 mg kg^−1^. However, samples containing* p*-CH_3_ at concentrations of 25 mg·kg^−1^ and 50 mg·kg^−1^ in combination with the PG at the concentration of 150 mg·kg^−1^ (codes CB-A3 and CB-B3) presented high IP values (8.31 ± 0.26 h and 8.25 ± 0.21 h, respectively). Thus, these results indicate that a synergistic effect between* p*-CH_3_ and PG is obtained when using low amount of* p*-CH_3_. An increase in the biodiesel stability in the blends containing* p*-CH_3_ with ALZ and CA, from 2.05 ± 0.45 (CB-A4) to 7.60 ± 0.22 (CB-C4); 4.90 ± 0.02 (CB-B5) to 7.97 ± 0.01 (CB-C5), respectively, was also observed. The combination between PG and ALZ (CB-D1 and CB-D5) also presented satisfactory synergism, with an increase in the IP values when compared to the results obtained by the PG (samples code: P-C1, P-C2, and P-C3, Table 4) used as individual additive (expected value higher than 8.0 h). The opposite was observed with the samples CB-D2, CB-D4, and CB-D6, suggesting absence of synergism when the PG is combined only with CA. For the combination of ALZ and CA, there is a slight positive effect, in which the IP values changed from 4.99 ± 0.05 h (PD-1, Table 4) and 3.76 ± 0.03 h (PE-1, Table 4)—for their individual addition—to 5.13 ± 0.28 h (CB-E1, Table 5) for their mixture.

In summary, the main characteristic of our findings is that an ideal blend can be obtained by mixing* p*-CH_3_ additive (at low concentration) with PG (a conventional antioxidant). This combination provides a synergistic effect in order to improve the biodiesel stability without raise the antioxidants' concentration [20].

### 3.3. Evaluation the Effectiveness of the Ternary Additive Blends

The ternary additive mixtures were evaluated and the results are shown in Table 6. In general, there was a positive effect based on the increase of the IP values with the addition of PG in the combination having the* p*-CH_3_ and CA or ALZ. As can be seen in Table 6, the IP values increased in proportion to the comparison from CT-A1 to CT-A6, CT-B1 to CT-B6, and CT-C1 to CT-C6. It is interesting to note that the most effective combinations were obtained by combining PG at 150 mg kg^−1^ and CA or ALZ at 20 mg kg^−1^ with* p*-CH_3_, independently of* p*-CH_3_ concentration. Moreover, the results also point out to a possible reestablishment of the capacity of the PG, promoted by the presence of* p*-CH_3_ and CA or ALZ, to stabilize biodiesel even after degradation induced by the presence of copper (e.g., comparison of CT-C4 with CT-C5 and CT-C6). The results also show that the samples CT-B6 and CT-C6 have similar IP values when compared with the binary sample CB-D5 (see Table 5), which was composed by PG at 150 mg kg^−1^ and ALZ at 20 mg kg^−1^. Therefore, from Rancimat test, it is possible to conclude that ternary blends did not present an appreciated synergetic effect induced by the presence of *p*-CH_3_ when compared with binary combinations.

### 3.4. Acid Compounds Formed during Oxidation Process

**Figure 2:**
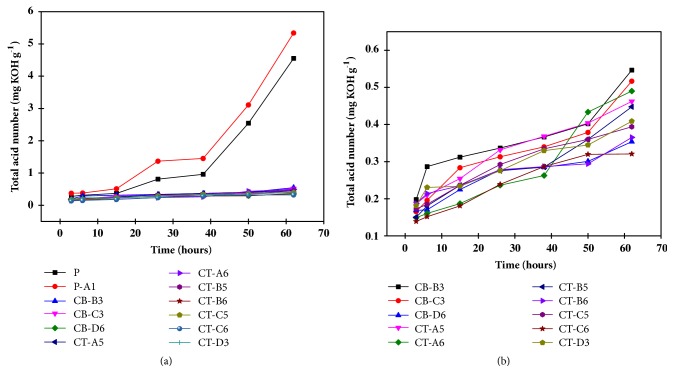
Total acid number of some selected representative samples heated at 85°C as a function of the heating time. (a) Selected representative samples. (b) Binary and ternary blends among the selected representative samples.

The formation of acid compounds is dependent of the different compositions of mixtures of the antioxidants as showed in Figure 2.  Figure 2(a) also shows that the pure biodiesel (P) presented a maximum value for total acid number (4.55 ± 0.15 mg KOH·g^−1^) at 62 h, indicating the high susceptibility to oxidation when subjected to heating under temperature at 85°C. The sample containing only copper (P-A1) reached a value of 5.34 ± 0.20 mg KOH·g^−1^ at 62h, which is higher (i.e., expected due to the presence of prooxidative metal) than the pure biodiesel. Thus, both samples exceeded the maximum acidity allowed by standard agencies (0.50 mg KOH·g^−1^) [11]. From Figure 2(b) it is possible to see that CB-B3 and CB-C3 combinations were able to maintain the TAN values less than 0.50 mg KOH·g^−1^ during the first 50 h of heating under temperature at 85°C. On the other hand, all other combinations presented TAN values below of the referenced value (0.50 mg KOH·g^−1^) up to the 62 h.

The sample (CT-C6) containing* p*-CH_3_ (100 mg·kg^−1^), PG (150 mg·kg^−1^), and ALZ (20 mg·kg^−1^), presented the lowest TAN value (0.32 ± 0.08 mg KOH·g^−1^) at 62 h of heating under temperature at 85°C. Despite the TAN results suggest that, on average, the ternary combinations can best avoid the formation of acid compounds, it is worth pointing out that binary mixtures were also powerful to stabilize biodiesel. For instance, the binary sample CB-D6 (containing 150 mg·kg^−1^ of PG and 20 mg·kg^−1^ of CA) presented a TAN value of 0.35 ± 0.05 mg KOH·g^−1^ at 62 h.

It is worth noticing that prooxidative metals are known to be present in petrodiesel and its blend with biodiesel can introduce metals-trace that may act as potent catalysts to accelerate the oxidation. Herein, the results showed that, when prooxidative metal is added to the biodiesel, the combinations having* p*-CH3, PG, and ALZ even at low concentration were potentially more effective in offering notable improvement in the biodiesel stability. Differently, the conventional antioxidant PG must be added individually at high concentration (e.g., 200 mg kg^−1^) to maintain the quality specifications of the biodiesel. As a consequence, it will be more costly to use a large amount of the standard antioxidant PG than the proposed alternative combination containing* p*-CH3, PG, and ALZ at low concentration for obtaining similar biodiesel preservation. In fact, the addition of high concentration of antioxidants into biodiesel will add value to the final product, increasing the final price paid by consumers.

### 3.5. Proposed Mechanism to Explain the Synergic Effect

**Figure 3:**
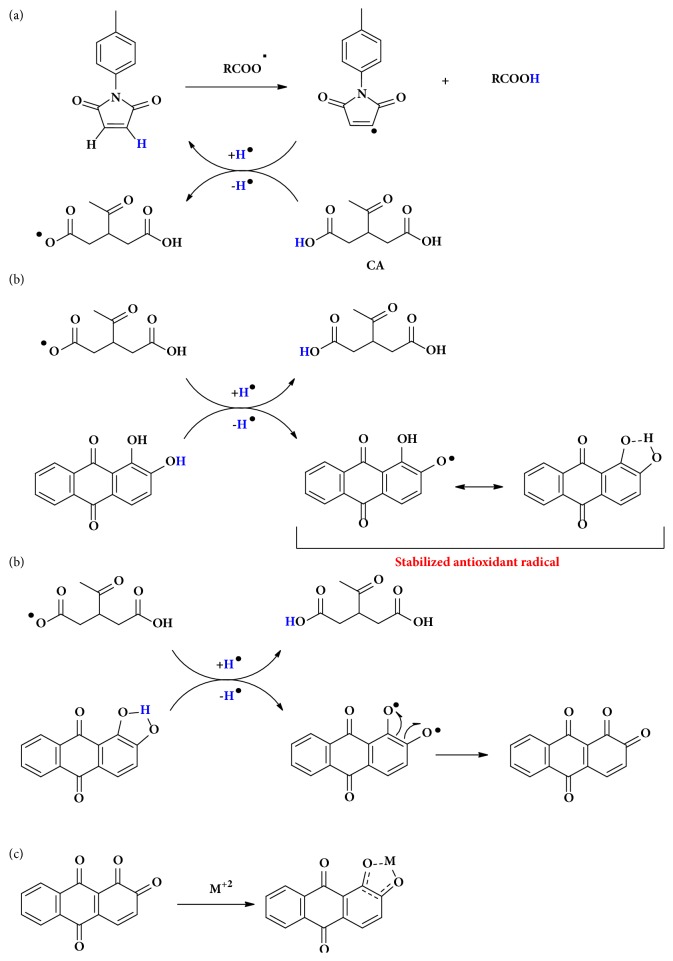
Proposed mechanism for the synergistic interactions between* p*-CH_3_, CA, and ALZ. (a) Cycle of regeneration of* p*-CH_3_, (b) cycle of regeneration of CA, and (c) chelation of diketone from ALZ with metals from metallic additives.

**Figure 4:**
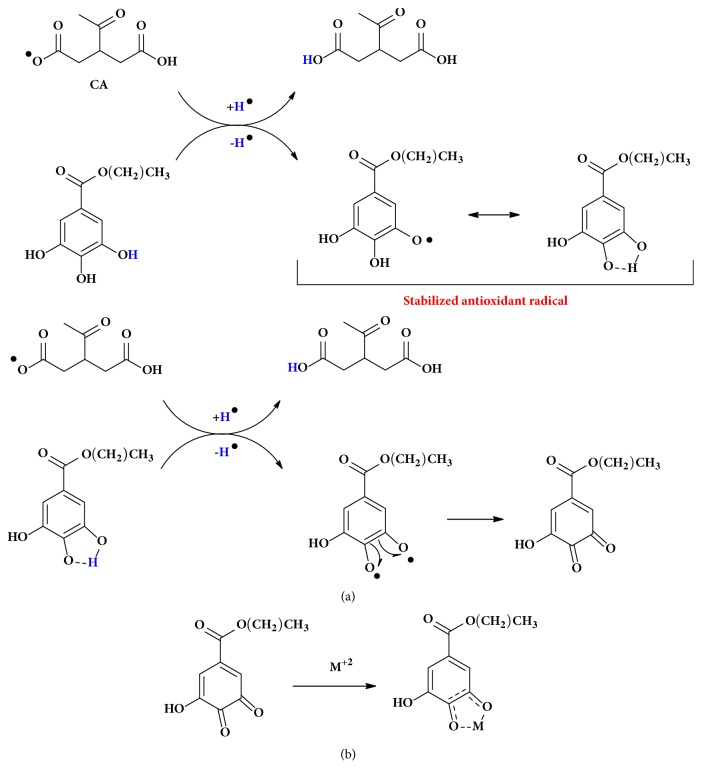
Proposed mechanism for the competitive synergistic interactions between p-CH_3_, CA, and PG. (a) Cycle of regeneration of CA and (b) chelation of diketone from PG with metals from metallic additives.

The suggested mechanism to explain the chemical reactions behind the multifunctional antioxidant activities and the synergetic effects claimed by suggested binary and ternary mixtures are shown in Figures 3 and 4, respectively. Here, the mechanism shows how target additives are able to improve the effectiveness of conventional antioxidants (PG) in stabilizing the biodiesel towards oxidation in presence of metal. The proposed mechanism (Figure 3) shows that the regeneration may happen through of a third additive, citric acid (CA). In this pathway, the CA becomes a hydrogen donor (i.e., pKa_1_ = 3.0, pKa_2_ = 4.7, pKa_3_ = 5.4) and, then, while the donor is consumed, the* p*-CH_3_ propagates its oxidation inhibition (Figure 3(a)). Then, the hydrogen donor CA will be regenerated by alizarin (ALZ) (pKa = 7.0), in which CA becomes able to donate one more hydrogen to the radical *p*-CH_3_. This mechanism shows that the hydrogen donation is favored because of to the stability of ALZ radical by resonance (Figure 3(b)). In the last step, the stabilization of CA happens because the radical from ALZ donates the second hydrogen and becomes a diketone, which in turn, can easily chelate with the Cu(II) (Figure 3(c)).

In that mechanism, the synergistic effect observed with both secondary antioxidants (ALZ and CA) occurs because ALZ can regenerate the CA, which can continue the regeneration of the primary antioxidant* p*-CH_3_. On the other hand, we propose a competitive synergistic interaction mechanism (Figure 4), which can allow the regeneration of the* p*-CH_3_ additive in a very similar way comparing to ALZ. In this case, the propyl gallate (PG) donates hydrogen to form the PG radical, which can be stabilized by resonance (Figure 4(a)). In a second step, the PG radical donates the second hydrogen generating a diketone which similarly to ALZ can be chelated by Cu(II) (Figure 4(b)). In this system (having* p*-CH_3_, ALZ, CA, and PG), we showed that the combination is useful, once it helps to maintain the activity of* p*-CH_3_ as inhibitor for the formation of fatty acid radicals, even at a low concentration and in the presence of prooxidative metal.

## 4. Conclusions

In the present study, it was showed the antioxidant potential of a novel additive,* p*-CH_3_, and the synergistic effect when combined with PG and ALZ or CA for stabilizing the biodiesel oxidation in the presence of Cu (II) as prooxidative metal. The results showed that the binary and ternary combinations presented synergetic effects to avoid biodiesel degradation, reaching the minimum requirements required by EN 14214. In addition, the results revealed that the binary mixture containing PG and p-CH_3_, even at low concentrations, can be successfully applied for biodiesel preservation, obtaining similar results observed to ternary blends (PG, p-CH_3_, and AC or AZL). Consequently, these results indicate that biodiesel stability can be obtained by using a reduced amount of additives combined in binary blends, demonstrating that biodiesel shelf life can be improved in association with a potential cost reduction when compared to the use of individual conventional antioxidants (e.g., PG).

## Figures and Tables

**Table 1:** Details of control and doped samples with different concentrations of additives.

Sample code	Additives and concentrations		
	Additive	Mass (mg·kg^−1^)	Cu(II)(mg·kg^−1^)
P	-	-	-
P- A1	-	-	2
P-B1	*p-CH*_*3*_	25	2
P-B2	*p*-CH_3_	50	2
P-B3	*p*-CH_3_	100	2
P-C1	PG	50	2
P-C2	PG	100	2
P-C3	PG	150	2
P-C4	PG	200	2
P-D1	ALZ	20	2
P-E1	CA	20	2

**Table 2:** Samples doped in binary combinations at different concentrations and the presence of Cu(II).

Sample code	Additives and concentrations			
	Additive 1	Mass (mg·kg^−1^)	Additive 2	Mass (mg·kg^−1^)
CB-A1	*p*-CH_3_	25	PG	50
CB-A2	*p*-CH_3_	25	PG	100
CB-A3	*p*-CH_3_	25	PG	150
CB-A4	*p*-CH_3_	25	ALZ	20
CB-A5	*p*-CH_3_	25	CA	20
CB-B1	*p*-CH_3_	50	PG	50
CB-B2	*p*-CH_3_	50	PG	100
CB-B3	*p*-CH_3_	50	PG	150
CB-B4	*p*-CH_3_	50	ALZ	20
CB-B5	*p*-CH_3_	50	CA	20
CB-C1	*p*-CH_3_	100	PG	50
CB-C2	*p*-CH_3_	100	PG	100
CB-C3	*p*-CH_3_	100	PG	150
CB-C4	*p*-CH_3_	100	ALZ	20
CB-C5	*p*-CH_3_	100	CA	20
CB-D1	PG	50	ALZ	20
CB-D2	PG	50	CA	20
CB-D3	PG	100	ALZ	20
CB-D4	PG	100	CA	20
CB-D5	PG	150	ALZ	20
CB-D6	PG	150	CA	20
CB-E1	ALZ	20	CA	20

**Table 3:** Samples doped in ternary combinations at different concentrations and the presence of Cu(II).

Sample code	Additives and concentrations					
	Additive 1	Mass	Additive 2	Mass	Additive 3	Mass
		(mg·kg^−1^)		(mg·kg^−1^)		(mg·kg^−1^)
CT-A1	*p*-CH_3_	25	PG	50	CA	20
CT-A2	*p*-CH_3_	25	PG	50	ALZ	20
CT-A3	*p*-CH_3_	25	PG	100	CA	20
CT-A4	*p*-CH_3_	25	PG	100	ALZ	20
CT-A5	*p*-CH_3_	25	PG	150	CA	20
CT-A6	*p*-CH_3_	25	PG	150	ALZ	20
CT-A7	*p*-CH_3_	25	ALZ	20	CA	20
CT-B1	*p*-CH_3_	50	PG	50	CA	20
CT-B2	*p*-CH_3_	50	PG	50	ALZ	20
CT-B3	*p*-CH_3_	50	PG	100	CA	20
CT-B4	*p*-CH_3_	50	PG	100	ALZ	20
CT-B5	*p*-CH_3_	50	PG	150	CA	20
CT-B6	*p*-CH_3_	50	PG	150	ALZ	20
CT-B7	*p*-CH_3_	50	ALZ	20	CA	20
CT-C1	*p*-CH_3_	100	PG	50	CA	20
CT-C2	*p*-CH_3_	100	PG	50	ALZ	20
CT-C3	*p*-CH_3_	100	PG	100	CA	20
CT-C4	*p*-CH_3_	100	PG	100	ALZ	20
CT-C5	*p*-CH_3_	100	PG	150	CA	20
CT-C6	*p*-CH_3_	100	PG	150	ALZ	20
CT-C7	*p*-CH_3_	100	ALZ	20	CA	20
CT-D1	PG	50	ALZ	20	CA	20
CT-D2	PG	100	ALZ	20	CA	20
CT-D3	PG	150	ALZ	20	CA	20

**Table 4:** Induction period (IP) for pure soybean biodiesel and containing individual additives obtained by Rancimat method. Sample code and concentration values are revealed in Tables 1–3.

Sample code	IP (hours)
P	3.76 ± 0.25
P- A1	0.20 ± 0.05
P-B1	0.80 ± 0.21
P-B2	1.05 ± 0.15
P-B3	1.20 ± 0.08
P-C1	5.04 ± 0.15
P-C2	6.75 ± 0.09
P-C3	7.49 ± 0.18
P-C4	3.40 ± 0.05
P-D1	4.99 ± 0.05
P-E1	3.76 ± 0.03

**Table 5:** Induction period (IP) for soybean biodiesel samples, containing binary additive blends, obtained by Rancimat method.

Sample	IP	Sample	IP	Sample	IP	Sample	IP
code	(hours)	code	(hours)	code	(hours)	code	(hours)
CB – A1	4.34 ± 0.01	CB – B1	4.32 ± 0.01	CB – C1	4.84 ± 0.10	CB – D1	8.46 ± 0.22
CB – A2	6.23 ± 0.13	CB – B2	4.73 ± 0.03	CB – C2	6.68 ± 0.05	CB – D2	4.94 ± 0.01
CB – A3	8.31 ± 0.26	CB – B3	8.25 ± 0.21	CB – C3	5.92 ± 0.12	CB – D3	4.34 ± 0.04
CB – A4	2.05 ± 0.45	CB – B4	2.46 ± 0.01	CB – C4	7.60 ± 0.22	CB – D4	6.23 ± 0.45
CB – A5	5.13 ± 0.11	CB – B5	4.90 ± 0.02	CB – C5	7.97 ± 0.01	CB – D5	8.31 ± 0.52
						CB – D6	1.92 ± 0.01
						CB – E1	5.13 ± 0.28

**Table 6:** Induction period (IP) for soybean biodiesel samples, containing ternary additive blends, obtained by the Rancimat® method.

Sample	IP	Sample	IP	Sample	IP	Sample	IP
code	(hours)	code	(hours)	code	(hours)	code	(hours)
CT – A1	6.25 ± 0.25	CT – B1	5.66 ± 0.06	CT – C1	5.75 ± 0.10	CT – D1	5.56 ± 0.02
CT – A2	5.47 ± 0.10	CT – B2	2.18 ± 0.01	CT – C2	2.00 ± 0.14	CT – D2	6.84 ± 0.23
CT – A3	7.80 ± 0.15	CT – B3	7.30 ± 0.15	CT – C3	6.74 ± 0.22	CT – D3	8.05 ± 0.08
CT – A4	6.87 ± 0.21	CT – B4	3.70 ± 0.02	CT – C4	2.83 ± 0.05	CT – D1	5.56 ± 0.01
CT – A5	8.68 ± 0.05	CT – B5	8.54 ± 0.12	CT – C5	8.96 ± 0.04		
CT – A6	8.14 ± 0.02	CT – B6	8.36 ± 0.03	CT – C6	8.35 ± 0.23		
CT – A7	4.43 ± 0.16	CT – B7	4.36 ± 0.01	CT – C7	4.34 ± 0.17		

## Data Availability

The data used to support the findings of this study are included within the article.
